# Similarly low blood metal ion levels at 10‐years follow‐up of total hip arthroplasties with Oxinium, CoCrMo, and stainless steel femoral heads. Data from a randomized clinical trial

**DOI:** 10.1002/jbm.b.35193

**Published:** 2022-11-10

**Authors:** Paul Johan Høl, Geir Hallan, Ove Furnes, Anne Marie Fenstad, Kari Indrekvam, Thomas Kadar

**Affiliations:** ^1^ Biomatlab, Department of Orthopedic Surgery Haukeland University Hospital Bergen Norway; ^2^ Department of Clinical Medicine University of Bergen Bergen Norway; ^3^ The Norwegian Arthroplasty Register, Department of Orthopedic Surgery Haukeland University Hospital Bergen Norway; ^4^ The Coastal Hospital at Hagevik, Department of Orthopedic Surgery Haukeland University Hospital Bergen Norway; ^5^ Physical Medicine and Rehabilitation, Clinic of Habilitation and Rehabilitation Haukeland University Hospital Bergen Norway

**Keywords:** arthroplasty, blood metal ion, chromium, cobalt, modular stem, oxidized zirconium, oxinium, RCT, trunnionosis

## Abstract

The use of inert head materials such as ceramic heads has been proposed as a method of reducing wear and corrosion products from the articulating surfaces in total hip arthroplasty, as well as from the stem‐head taper connection. The aim of the present study was to compare the blood metal ion levels in patients with Oxinium and CoCrMo modular femoral heads, as well as monoblock stainless steel Charnley prostheses at 10 years postoperatively. The 150 patients with osteoarthritis of the hip joint included in a randomized clinical trial were grouped according to femoral head material. One group (*n* = 30) had received the Charnley monoblock stainless steel stem (DePuy, UK). The other patients (*n* = 120) received a Spectron EF CoCrMo stem with either a 28 mm CoCrMo or Oxinium modular head (Smith & Nephew, USA). After 10 years, 38 patients had withdrawn, 19 deceased, 7 revised due to aseptic loosening and 5 revised due to infection. The 81 patients with median age of 79 years (70–91) were available for whole blood metal ion analysis. The levels of Co, Cr, Ni and Zr in the blood were generally low with all the head materials (medians <0.3 micrograms/L) and no statistical difference between the groups were found (*p* = .2–.8). Based on the low blood metal ion values in our study groups, no indication of severe trunnion corrosion in patients with CoCrMo heads was observed, neither was there any beneficial reduction in metal ion exposure with the Oxinium femoral heads.

## 1 INTRODUCTION

The occurrence of wear and corrosion at the trunnion‐head interface (trunnionosis) has gained wider recognition as an important clinical matter in metal‐on‐metal^1^, ^2^, ^3^ as well as metal‐on‐polyethylene total hip arthroplasty (THA).^4^, ^5^ Some suggest that trunnionosis accounts for up to 3% of all THA failures.^6^, ^7^ Apart from aseptic loosening and osteolysis from local release of metal debris, also systemic toxicity related to high cobalt ion levels with neurological^8^ and cardiac^9^, ^10^ manifestations (termed cobaltism), has caused further concerns.

Oxidized zirconium (Oxinium™) femoral heads were introduced with the prospects of combining the strength of a metal head with the smoothness and hardness of ceramic.^11^ Oxinium has a metal alloy core (zirconium‐niobium) and an approximately 5 μm thick zirconia ceramic surface made by heat treatment of the surface.^12^ Theoretically, the Oxinium femoral head and taper junction can reduce the release of metals due to the ceramic surface. One study showed that the use of Oxinium femoral head decreased the blood cobalt levels versus a cobalt‐chromium‐molybdenum alloy (CoCrMo) femoral head, albeit it did not reach statistical significance.^13^ However, a retrieval study showed no difference in the corrosion score between Oxinium and CoCrMo femoral heads.^14^

In this randomized clinical study, we wanted to evaluate the blood metal ion levels of Oxinium modular femoral heads at 10 years postoperatively and compare this with patients with the CoCrMo modular femoral head counterpart, and the monoblock stainless steel Charnley prosthesis. The null hypothesis was that blood metal ion levels with Oxinium femoral heads were equal to that of the metal heads.

## 2 PATIENTS AND METHODS

Between November 2004 and June 2007, 150 patients with primary or secondary osteoarthritis of the hip were included in a randomized study investigating polyethylene wear of five different articulations and migration of the implants. The randomization, surgical procedure, post‐operative treatment and method of wear measurement has previously been described in detail.^15^, ^16^

**FIGURE 1:**
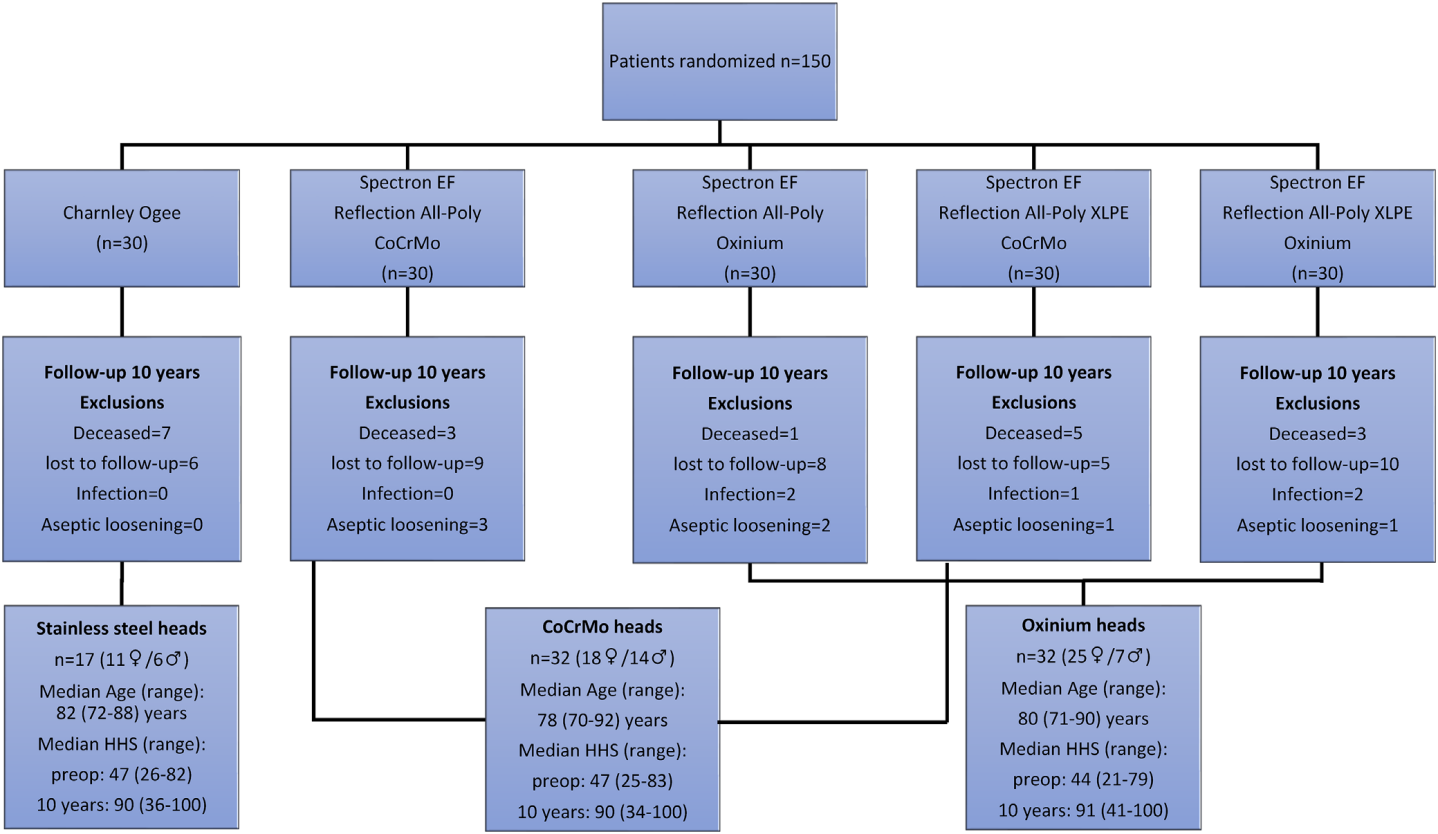
Participant flow diagram describing the original five study groups based on the different head‐cup articulations and the final three groups based on head material (stainless steel, CoCrMo, and Oxinium). Exclusions after 10 years follow‐up are sorted after revision causes (infection or aseptic loosening) and reasons for drop out (deceased or lost to follow‐up). The gender distribution, age and Harris Hip Score (median and range) in the three study groups are specified

**FIGURE 2:**
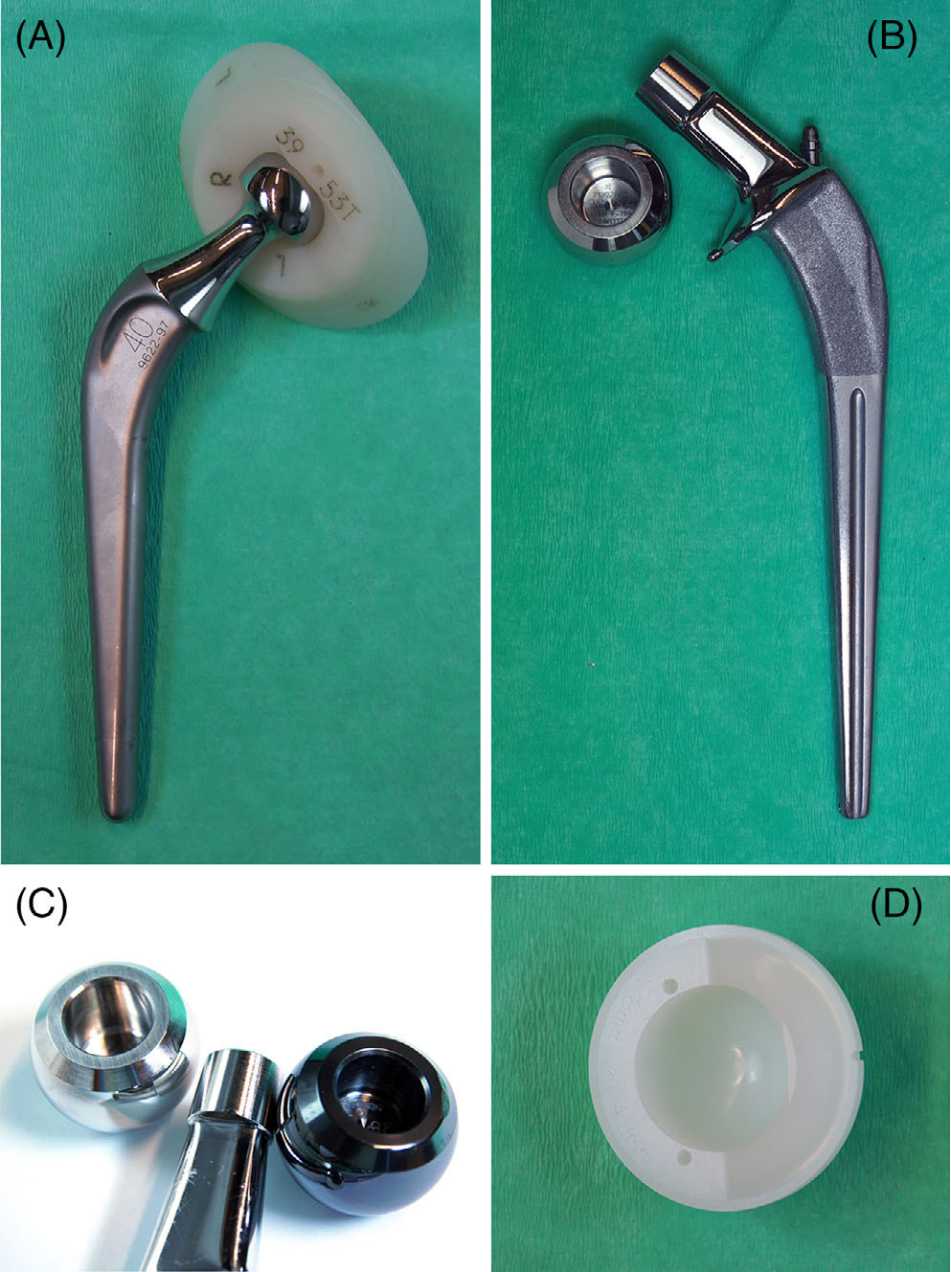
(A) Charnley flanged 40 monoblock, oval, 316 L stainless steel, matte surface (Ra 0.8 μm) femoral stem with a 22.2 mm head, which articulated with a Charnley Ogee UHMWPE acetabular cup. (B) Spectron EF CoCrMo femoral stem with a 28 mm CoCrMo modular femoral head, retrieved from study case n° 819, with abrasive marks on the stem‐cement interface, mainly on the posterior‐distal‐medial side. The collared Spectron EF stem was fabricated with a matte distal surface (Ra 0.7 μm) and a grit blasted roughened surface (Ra 7.3 μm) on the proximal third of the stem. The neck and collar are highly polished.^15^ The stem used in this RCT was supplied with three tantalum markers, one of which was attached to a cone in the neck, the second in the medial side of the collar and the third on the distal tip of the stem. (C) CoCrMo 28 mm femoral head (left) and Oxinium 28 mm femoral head (right). Spectron EF stem taper size 12/14, standard offset (in the middle). (D) Reflection All‐Poly UHMWPE cup.

The patients were randomized into five groups of cemented THAs with *n* = 30 in each group (Figure 1). One group received the Charnley flanged 40 femoral 316 L stainless steel monoblock stem with a 22.2 mm head, which articulated with a Charnley Ogee UHMWPE acetabular cup (DePuy, UK) (Figure 2A). The other four groups received a Spectron EF CoCrMo femoral stem (Figure 2B) with either a 28 mm CoCrMo or a 28 mm Oxinium femoral head (Figure 2C), which articulated with either an ethylene oxide‐sterilized Reflection All‐Poly UHMWPE cup or a Reflection All‐Poly highly cross‐linked polyethylene (XLPE) cup (Smith&Nephew, UK) (Figure 2D). The sizes of the Spectron EF stems varied between size 2–5 (length/distal cross section [mm]: 125/7–135/12, neck angle: 131°). All Spectron EF stems had standard offset and the taper junctions possessed a 12/14 geometry with a ridged male taper surface (Figure 2C).

Exclusion criteria were BMI > 35, uncompensated cardio‐pulmonary disease, malignant disease, dementia, rheumatoid arthritis or other serious systemic diseases.

Of the 150 patients in the original study, 19 patients had deceased and 38 patients withdrew from the study and could therefore not provide a blood sample at 10 years follow‐up (not able or willing to participate) (Figure 1). Seven patients were revised before 10 years follow‐up due to aseptic loosening of either the cup or stem or both. 4 of these cases was included in a retrieval project where explants were collected with periprosthetic tissue and whole blood samples.^17^ Additionally, five hips were revised due to infection. None of the patients in the Charnley group were revised.

After 10‐year follow‐up, the remaining 81 patients (54 females and 27 men from the original five groups were regrouped into three groups, depending on the femoral head material used at the time of surgery) (Figure 1). The overall median age were 79 years (70–91). At 10 years follow‐up we found that many patients had received prostheses in other joints, based on information from the Norwegian Arthroplasty Register (see further details in supplement 1).

### 2.1 Blood metal ion analysis

Whole blood was drawn from a vein in the forearm using an intravenous catheter (venesection) in our hospital (Becton Dickinson Venflon Pro™, 1.5 × 45 mm, Helsingborg, Sweden). After venipuncture, the steel needle was removed, and the blood (5–10 ml) was allowed to flow directly into a 15 ml metal free polypropylene tube (VWR International, CS500, Radnor, PA, US). The blood was stored at minus 20°C until further handling. The concentration of chromium, cobalt, nickel and zirconium in whole blood was determined by Inductively Coupled Plasma–Mass Spectrometry (ICP‐MS). The instrument (Element XR™ HR‐ICP‐MS, Thermo Fisher Scientific, Bremen, Germany) was equipped with a high resolution, magnetic sector field. Before analysis, an aliquot of approximately 1.5 g whole blood was acid digested by adding 3 ml 60% ultrapure HNO_3_ and 2 ml 30% H_2_O_2_ (Merck, Darmstadt, Germany) in closed vessels in a microwave‐assisted system (Milestone 1200 Mega, Sorisole, Italy). After digestion the samples were diluted approximately 66 times with double distilled deionized water (MilliQ, USA). Calibration was performed by standard addition using 0.2, 0.5, 2 and 5 μg/L calibration solutions prepared from a 10 μg/mL multi‐element stock standard solution (Spectrascan, Lot Number C2‐MEB294066, Teknolab AB, Kungsbacka, Sweden). An internal standard of indium was injected automatically to all the samples to monitor and correct for any instrumental changes. The method detection limit (MDL) for element analysis was defined as three times the standard deviation in 10 blank solutions measured at different times, taking the dilution into account. The MDL was 0.11 μg/L for Cr, 0.04 μg/L for Co, 0.21 for Ni and 0.02 for Zr. The accuracy of the analytical method was monitored using a reference material (Seronorm Trace Elements Whole Blood L‐2, lot 1,103,129, Sero AS, Billingstad, Norway).

### 2.2 Statistics

All statistical analyses were performed using the IBM SPSS Statistics for Macintosh, Version 27.0 (IBM Corp, Armonk, NY, USA). The graph was made with GraphPad Prism software, version 9.3.1 (GraphPad Software Inc., San Diego, CA, USA). Nonparametric statistical methods were chosen since the metal concentrations in the study groups had a skewed distribution and did not pass the normality test. Kruskal–Wallis one‐way analysis of variance (two‐sided) was used to detect differences in average blood ion levels among the three study groups. Additionally, the metal ion levels in the original study groups with five different articulations were compared with Kruskal‐Wallis test to see if there were any influence of the cup materials. An independent samples Mann–Whitney *U* test (two‐sided) was used to compare metal ion levels between patients with prostheses in other joints (pooled) versus patients with the study prosthesis only (pooled). Clinical outcome was evaluated with the Harris Hip Score (HHS). Median group HHS scores are reported preoperatively and at 10 years (Figure 1) and study groups statistically compared with Kruskal–Wallis one‐way analysis of variance (two‐sided). Probability (*p*) values of <.05 denote statistical significance.

## 3 RESULTS

Patients in the stainless steel, CoCrMo and Oxinium femoral head groups demonstrated significantly higher HHS scores 10 years postoperatively compared to preoperative scores, and there were no significant differences in clinical function between the three groups (*p* = .69) (Figure 1).

**FIGURE 3:**
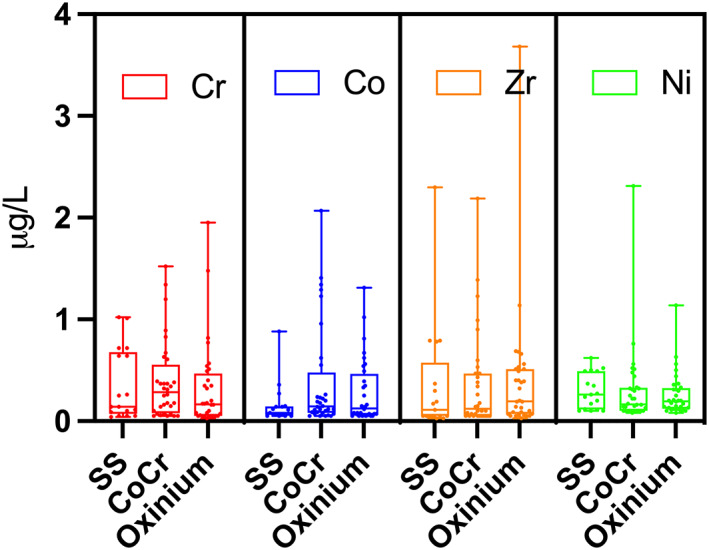
Boxplot of blood metal ion levels (μg/L) for Cr, Co, Zr, and Ni, grouped on head material. Horizontal lines inside the boxes indicates median level. Whiskers represents min‐max values (range). SS, stainless steel

The median blood metal ion levels for Cr, Co, Zr, and Ni were generally low in all study groups (Table 1 and Figure 3). Patients with the modular CoCrMo head had approximately twice as high median Cr blood concentration (0.29 μg/L) than the Oxinium group (0.17 μg/L) and the nonmodular stainless steel group (0.14 μg/L). The median Co level for the CoCrMo head group (0.15 μg/L) was almost twice the median level (0.08 μg/L) for the stainless steel group, but similar to the Oxinium group (0.15 μg/L). The median zirconium level was highest in the Oxinium group (0.2 μg/L), actually 4 times higher than in the stainless steel group. Still, we were not able to find statistical differences in any of the blood metal ion levels between the three head groups (*p* = .2–.8) (Table 1). The different acetabular cup materials (conventional or highly crosslinked polyethylene) did not influence the metal levels (*p* = .2–.9, five groups).

**TABLE 1:** Cr, Co, Zr and Ni concentrations in the three groups based on head material (stainless steel, CoCrMo and Oxinium), represented with median, mean and maximum (max) values (microgram/L). Statistical difference between the groups is calculated by Kruskal‐Wallis one‐way test

	Cr	Co	Zr	Ni
Stainless steel heads (*n* = 17)				
Median	0.14	0.08	0.05	0.24
Mean	0.38	0.25	0.32	0.29
Max	1.02	1.96	2.30	0.62
CoCrMo heads (*n* = 32)				
Median	0.29	0.15	0.12	0.17
Mean	0.39	0.39	0.36	0.31
Max	1.52	2.07	2.19	2.31
Oxinium heads (*n* = 32)				
Median	0.17	0.13	0.20	0.20
Mean	0.33	0.28	0.41	0.26
Max	1.95	1.31	3.68	1.14
*p* values (Kruskal‐Wallis)	.44	.25	.18	.81

Seven patients were revised due to aseptic loosening of either the cup or stem or both. None of the patients in the Charnley group were revised. See table 2 for revision cause and blood metal levels. One of the Spectron cases with CoCrMo head that was revised after 8 years (study *n*° 819) had the highest Co level (5.8 μg/L) among all the patients in the study. The Co level was substantially higher than the Cr level (0.76 μg/L). We could not detect any visible taper corrosion on the revised stem under the stereomicroscope. However, we found extensive polishing and abrasive marks on the stem‐cement interface, especially at the anterior‐distal‐lateral and the posterior‐distal‐medial stem corners (Figure 2B).

**TABLE 2:** Patients that were revised before 10 years follow‐up due to aseptic loosening of either the cup or stem or both. Three of these cases was included in a retrieval project and whole blood samples collected and the concentration (microgram/L) of Cr, Co, Ni, and Zr measured (study no 819, 831, and 848)

Study n°	Head group	Revised component	Years in vivo	Cr μg/L	Co μg/L	Ni μg/L	Zr μg/L
819	CoCr head/eto‐PE	Cup and stem	8	0.76	5.8	0.63	1.94
831	Ox head/eto‐PE	Cup and head	6	0.82	0.34	0.74	0.5
848	Ox head/eto‐PE	Cup and head	8	0.37	0.16	NA	<DL
857	CoCr head/eto‐PE	Cup and stem	7	NA	NA	NA	NA
805	CoCr head/XLPE	Cup and stem	9	NA	NA	NA	NA
804	Ox head/XLPE	Cup	6	NA	NA	NA	NA
844	CoCr head/eto‐PE	Cup	10	NA	NA	NA	NA

Many study patients had prostheses in other joints, surgery performed either before or within the study period (55 of 81), see Supplement 1 for further details. 50 patients had a contralateral hip replacement. The femoral stems used in the other hip was the Lubinus SP II (*n* = 22), Spectron EF (*n* = 12), Charnley (*n* = 6), Exeter (*n* = 3), Profile (*n* = 3), Titan, Corail, Elite and MS‐30 (*n* = 1). 12 patients had a total knee replacement; Profix (*n* = 5), NexGen (*n* = 2), LCS Complete (*n* = 3) and Genesis (*n* = 2). The four of these had bilateral knee replacement. Four patients also had shoulder prosthesis. The patient group with Oxinium femoral heads had the highest portion of prostheses in other joints (23 of 32), followed by the Charnley stainless steel group (12 of 17) and the CoCrMo group (20 of 32). In patients who had additional prostheses (*n* = 55) the median metal ion levels were not statistically different (*p* = .2–.9) to those with only the study prosthesis (*n* = 26) (Supplement 2).

## 4 DISCUSSION

In the present study of primary THAs we found no difference in blood ion levels with the use of Oxinium femoral heads compared to CoCrMo or stainless steel heads at 10 years follow‐up. To our knowledge this is the first RCT who compares metal ion release from these three femoral head materials.

All the Co and Cr values were well below the British Medicines and Healthcare Products Regulatory Agency threshold value of 7 μg/L. All the median Co and Cr values for the three head materials were similar and all below 0.3 μg/L. This is actually comparable with preoperative blood metal values measured with the same methodology as in our recent study, where we found median Cr and Co concentrations of 0.23 μg/L and 0.14 μg/L, respectively, in a cohort of patients measured prior to receiving Birmingham hip resurfacing prostheses.^18^ It is also comparable with preoperative serum metal concentrations to another study, where they found mean Cr and Co concentrations of 0.3 μg/L and 0.2 μg/L, respectively.^2^ The same patients received metal‐on‐polyethylene THAs with modular 28 mm CoCrMo femoral heads, and after 16 years follow‐up, mean serum Cr and Co levels increased to 1.0 μg/L (95% CI: 0.7–1.4 μg/L) and 0.4 μg/L (95% CI: 0.1–0.6 μg/L), respectively.

The mechanisms of trunnionosis are currently poorly understood and is likely multi‐factorial.^19^ Several Electrochemical and mechanical factors have been suggested to increase the risk. Implant‐based factors that have been postulated to affect the degree of trunnionosis include length and diameter of the head, the dimensions of the trunnion, the surface finish and flexural rigidity of the trunnion and the head‐trunnion interface materials.^4^, ^20^ Patients with CoCr‐containing THAs had more necrosis and lymphocytic infiltration in their tissues than patients with hip resurfacings, indicating that trunnion wear debris produced more cytotoxic and immunogenic reactions than bearing wear debris.^21^ Especially the cobalt species has been under suspicion for its adverse effects, such as cardiac toxicity.^9^ However, recent data from the National Joint Registry and NHS English hospital inpatient episodes about CoCr‐containing THAs, did not find increased risk of all‐cause mortality, or clinically meaningful heart outcomes, cancer, or neurodegenerative disorders into the second decade post‐implantation.^22^

One study has suggested that by using a ceramic femoral head, CoCrMo tribocorrosion may be mitigated.^23^ Two other studies also concluded that ceramic head confers an advantage in trunnion fretting and corrosion.^14^, ^24^

Mid‐term in vivo wear results (2‐ and 5‐year follow‐up based on radiostereometric analysis [RSA]) have not shown wear benefits of Oxinium femoral heads over conventional CoCrMo femoral heads.^11^, ^16^ There are reports that Oxinium femoral heads are susceptible to damage upon hip dislocation with subsequent increased polyethylene wear.^25^ A recent case report showed catastrophic failure of an Oxinium‐coated total knee prosthesis (Smith and Nephew) that resulted in metallosis with extra‐articular extravasation.^26^ In a systematic review and meta‐analysis, Oxinium femoral heads did not lead to lower polyethylene wear rate or higher survival rate, when compared with CoCrMo femoral heads in patients treated with THA.^27^ In a prospective study comparing whole blood Cr, Co and Ti ion concentrations in young, active patients (N = 36) undergoing primary THA with uncemented TiAlV stems and CoCrMo‐alloy (32 mm), ceramic (32–40 mm), or Oxinium (32 and 36 mm) femoral heads, no statistically significant differences were found between the three cohorts, between 1 and 5 years follow‐up (*p* = .3–.5).^13^ The evidence of any benefits with the use of Oxinium femoral heads is therefore still sparse and further investigation regarding any benefit with Oxinium femoral heads is therefore needed. That said, clinical data from the Australian and UK registry indicate excellent results for Oxinium used in combination with well‐performing cup and stems.^28^, ^29^

Furthermore, it is now well established that highly cross‐linked polyethylene acetabular cups and liners significantly reduces wear compared to conventional polyethylene.^30^ This has allowed for the use of larger head sizes to improve prosthetic stability. However, larger femoral head sizes may increase stress at the modular head–neck junction, increasing the effective horizontal lever arm, potentially leading to fretting corrosion and metal debris with the use of cobalt‐alloy femoral heads.^24^, ^31^ In this study only small head sizes were used. Thus, we cannot deduct our results to larger head sizes. However, in a recent study studying metal ion levels with modular 28 mm, 36 mm and 40 mm CoCrMo femoral heads, no difference in cobalt and chromium levels were found with increasing femoral head sizes after 10 years follow‐up.^32^ The median cobalt and chromium levels for the 28 mm head group were 0.66 μg/L and 0.68 μg/L, respectively. Similarly, no difference was found in cobalt and chromium blood levels in a randomized study comparing 32 mm, 36 mm and 40 mm modular CoCrMo heads at 1‐ and 2‐years follow‐up.^33^

Neck taper flexural rigidity also affect trunnion corrosion and one study showed that lower stem flexural rigidity predicted stem fretting and corrosion damage in a ceramic head cohort, but not in the metal head cohort.^23^ Care should therefore be taken to generalize the results to different stem designs and materials.

### 4.1 Strengths and limitations

A limitation of this study is the relatively small cohort of 64 patients with modular femoral implants. It might not be adequate to characterize a relatively infrequent condition like trunnion corrosion. The size of our cohorts contributed to the large interquartile range seen in metal ion levels, and despite difference in some median levels between the head material groups, these failed to reach statistical difference.

Furthermore, we did not have a control group of patients with native hip, without implants. Yet, the monoblock stainless steel Charnley prosthesis had similar metal ion levels as the modular Spectron EF prosthesis, regardless of femoral head material. Trunnionosis cannot account for the metal ion levels with the monoblock Charnley, showing that systemic metal ions may stem from other sources than the trunnion. The Spectron EF femoral stem was more stable than the Charnley stem in a RSA‐study when evaluated at 2 years.^15^ Theoretically the blood ion levels with the Charnley prosthesis may be the result of corrosion at the stem‐cement interface and from the articulation (metal head). Analysis of the retrieved prostheses has been undertaken to clarify this matter in another study. Cases with Spectron EF stem loosening had significantly higher median Cr (1.05 μg/L), Co (1.85 μg/L), and Zr (0.65 μg/L) concentrations in blood at the day of revision surgery compared to the unrevised patients in this study, with probably still well‐fixed Spectron EF stems.^17^ It was concluded that fretting wear of loose stems against the cement mantle may explain the elevated metal ion levels, where Co and Cr originate from the stem and Zr from the bone cement. It is interesting to note that cases with cup and head revision only had relatively low blood metal values, comparable with the unrevised patients at 10 years in this study. On the other side, one case with both stem and cup loosening (study n° 819), had more elevated Co (5.8 μg/L), and Zr (1.94 μg/L) values (Table 2). This is most likely attributed to fretting wear of the loose stem against the abrasive ZrO_2_ containing cement mantle (Figure 2B).

Our results highlight the importance of retrieval centers for understanding cause and effect relationships in THA.^17^, ^34^ An interesting research question for a future study of our study patients is to investigate if there is an association between implant migration measured by radiostereometry and blood metal ion levels as an indicator of stem loosening.

Another limitation with the study is that 50 patients had hip prosthesis in the other hip. Some patients also had prostheses in the knee (*n* = 12) and in the shoulder (*n* = 4), see Supplement 1 for details about implant brands. This can contribute extra to the total metal burden in the blood and might hide differences between the study groups, although this does not seem to be the case, since individuals who only had the study prosthesis did not have significantly lower metal levels (supplement 2). Despite potential for metal contamination from other implants, the metal levels were low in all groups, ruling out there is a serious problem with metal ion release due to trunnionosis with our study prostheses at 10 years post‐op.

One strength of this study is that it was part of a randomized study, reducing the risk of selection bias. To our knowledge this is the first RCT who compares metal ion release from the femoral head materials Oxinium, CoCrMo and stainless steel. Another strong point of this study is that the taper design was identical between the CoCrMo and Oxinium modular head groups. Taper design is associated with the degree of crevice corrosion at the modular head and neck interface. Our results are therefore solely associated with head–neck material, not variance in taper design.

Detecting differences in blood ion levels between groups is demanding due to small detection limits.^35^ In the present study a single laboratory, with a highly sensitive methodology and adequate detection limits, analyzed all blood samples. This allows more subtle differences between groups to be detected.

Our study patients were quite old, so our results may not apply to younger and more active patients, who are increasingly becoming eligible for the THA procedure. The mechanical demands at the head‐trunnion in a younger population interface may be higher. Thus, any mechanically driven corrosion and rise in blood ion levels may be underestimated in the population of this study.

## 5 CONCLUSION

In this study of THAs with small femoral heads, no indication of severe trunnion corrosion was found. Although we found no significant difference in blood ion levels with the use of Oxinium or metal femoral heads, further investigation of periprosthetic tissues and retrieved implants may give insight to any differences in wear and corrosion mechanism at the trunnion‐head interface between CoCrMo and Oxinium femoral heads.

## AUTHOR CONTRIBUTIONS

Paul Johan Høl: Collected and interpreted the data, performed the statistical analysis, created the figures, carried out literature search, wrote the article and revised the manuscript.

Thomas Kadar: Designed the study, provided clinical review of patients in clinic, wrote the article, carried out literature search.

Geir Hallan, Ove Furnes, and Kari Indrekvam: Designed the study, revised the manuscript.

Anne Marie Fenstad: Provided patient details from initial study, controlled the statistical analysis and revised the manuscript.

## FUNDING INFORMATION

The study was supported financially by Western Norway Regional Health Authority, Smith & Nephew Norway and Ortomedic AS. None of the sources of funding were involved in the planning or analysis of results.

## CONFLICT OF INTEREST

The authors declare that they have no competing interests.

## Supporting information


**Appendix S1:** Supplement 1. Overview of included study patients (n = 81) with their study prosthesis (femoral stem, head and cup) grouped by femoral head material. Further columns list additional hip stems (SS = stainless steel), knee and shoulder prostheses implanted either before or within the 10‐year study period.

**Appendix S2:** Supplement 2. A comparison of the of the blood metal levels (μg/l) in patients with only the study prosthesis (pooled) and those with additional prostheses (pooled). An independent samples Mann–Whitney U test was used to compare ranks.

## Data Availability

The data that support the findings of this study are available upon request from the corresponding author.
