# Supplementary material for: Similarly low blood metal ion levels at 10‐years follow‐up of total hip arthroplasties with Oxinium, CoCrMo, and stainless steel femoral heads. Data from a randomized clinical trial
Source: J Biomed Mater Res B Appl Biomater. 2022 Nov 10;111(4):821–8. doi: 10.1002/jbm.b.35193 (PMC10099800; doi:10.1002/jbm.b.35193)
Supplement: Supplementary file 1 — Appendix S1: Supplement 1. Overview of included study patients (n = 81) with their study prosthesis (femoral stem, head and cup) grouped by femoral head material. Further columns list additional hip stems (SS = stainless steel), knee and shoulder prostheses implanted either before or within the 10‐year study period. [file JBM-111-821-s001.docx]

**Supplement 1.** Overview of included study patients (n=81) with their study prosthesis (femoral stem, head and cup) grouped by femoral head material. Further columns list additional hip stems (SS=stainless steel), knee and shoulder prostheses implanted either before or within the 10-year study period.

| **Patient** | **Stem brand** | **Head material** | **Cup brand and material** | **2nd hip**  **(stem material)** | **1st knee brand (material)** | **2nd knee brand (material)** | **Shoulder brand (material)** |
| --- | --- | --- | --- | --- | --- | --- | --- |
| 1 | Charnley flanged 40 | **Stainless steel, 316L** | Charnley Ogee UHMWPE | Lubinus SP II (CoCrMo) |  |  |  |
| 2 | Charnley flanged 40 | **Stainless steel, 316L** | Charnley Ogee UHMWPE | Profile (TiAlV) | Genesis I (CoCr/TiAlV) |  |  |
| 3 | Charnley flanged 40 | **Stainless steel, 316L** | Charnley Ogee UHMWPE |  |  |  |  |
| 4 | Charnley flanged 40 | **Stainless steel, 316L** | Charnley Ogee UHMWPE |  |  |  |  |
| 5 | Charnley flanged 40 | **Stainless steel, 316L** | Charnley Ogee UHMWPE | Charnley (SS) | Profix (CoCrMo) | Profix (CoCrMo) |  |
| 6 | Charnley flanged 40 | **Stainless steel, 316L** | Charnley Ogee UHMWPE | Lubinus SP II (CoCrMo) |  |  |  |
| 7 | Charnley flanged 40 | **Stainless steel, 316L** | Charnley Ogee UHMWPE |  | Profix (CoCrMo) |  |  |
| 8 | Charnley flanged 40 | **Stainless steel, 316L** | Charnley Ogee UHMWPE | Lubinus SP II (CoCrMo) |  |  |  |
| 9 | Charnley flanged 40 | **Stainless steel, 316L** | Charnley Ogee UHMWPE | Lubinus SP II (CoCrMo) |  |  |  |
| 10 | Charnley flanged 40 | **Stainless steel, 316L** | Charnley Ogee UHMWPE | Lubinus SP II (CoCrMo) |  |  |  |
| 11 | Charnley flanged 40 | **Stainless steel, 316L** | Charnley Ogee UHMWPE |  | LCS (CoCrMo) |  |  |
| 12 | Charnley flanged 40 | **Stainless steel, 316L** | Charnley Ogee UHMWPE |  |  |  |  |
| 13 | Charnley flanged 40 | **Stainless steel, 316L** | Charnley Ogee UHMWPE |  |  |  |  |
| 14 | Charnley flanged 40 | **Stainless steel, 316L** | Charnley Ogee UHMWPE | Elite (CoCrMo) |  |  |  |
| 15 | Charnley flanged 40 | **Stainless steel, 316L** | Charnley Ogee UHMWPE | Lubinus SP II (CoCrMo) |  |  |  |
| 16 | Charnley flanged 40 | **Stainless steel, 316L** | Charnley Ogee UHMWPE | Lubinus SP II (CoCrMo) |  |  |  |
| 17 | Charnley flanged 40 | **Stainless steel, 316L** | Charnley Ogee UHMWPE |  |  |  |  |
| 18 | Spectron EF | **CoCrMo** | Reflection All-Poly UHMWPE |  |  |  |  |
| 19 | Spectron EF | **CoCrMo** | Reflection All-Poly UHMWPE | Lubinus SP II (CrCoMo) | LCS (CoCrMo) |  |  |
| 20 | Spectron EF | **CoCrMo** | Reflection All-Poly UHMWPE |  |  |  |  |
| 21 | Spectron EF | **CoCrMo** | Reflection All-Poly UHMWPE | Lubinus SP II (CoCrMo) | Profix (CoCrMo) | Profix (CoCrMo) |  |
| 22 | Spectron EF | **CoCrMo** | Reflection All-Poly UHMWPE |  |  |  |  |
| 23 | Spectron EF | **CoCrMo** | Reflection All-Poly UHMWPE | Lubinus SP II (CoCrMo) |  |  |  |
| 24 | Spectron EF | **CoCrMo** | Reflection All-Poly UHMWPE | Lubinus SP II (CoCrMo) |  |  |  |
| 25 | Spectron EF | **CoCrMo** | Reflection All-Poly UHMWPE | Spectron-EF (CoCrMo) |  |  |  |
| 26 | Spectron EF | **CoCrMo** | Reflection All-Poly UHMWPE | Spectron-EF (CoCrMo) |  |  |  |
| 27 | Spectron EF | **CoCrMo** | Reflection All-Poly UHMWPE |  |  |  |  |
| 28 | Spectron EF | **CoCrMo** | Reflection All-Poly UHMWPE | Lubinus SP II (CoCrMo) |  |  |  |
| 29 | Spectron EF | **CoCrMo** | Reflection All-Poly UHMWPE |  |  |  |  |
| 30 | Spectron EF | **CoCrMo** | Reflection All-Poly UHMWPE | Lubinus SP II (CoCrMo) |  |  |  |
| 31 | Spectron EF | **CoCrMo** | Reflection XLPE |  |  |  |  |
| 32 | Spectron EF | **CoCrMo** | Reflection XLPE | Lubinus SP II (CoCrMo) |  |  |  |
| 33 | Spectron EF | **CoCrMo** | Reflection XLPE |  |  |  | Global Adv. (CoCrMo) |
| 34 | Spectron EF | **CoCrMo** | Reflection XLPE | Spectron-EF (CoCrMo) |  |  |  |
| 35 | Spectron EF | **CoCrMo** | Reflection XLPE | Charnley (SS) |  |  |  |
| 36 | Spectron EF | **CoCrMo** | Reflection XLPE |  |  |  |  |
| 37 | Spectron EF | **CoCrMo** | Reflection XLPE | Spectron-EF (CoCrMo) |  |  |  |
| 38 | Spectron EF | **CoCrMo** | Reflection XLPE |  |  |  |  |
| 39 | Spectron EF | **CoCrMo** | Reflection XLPE | Lubinus SP II (CoCrMo) |  |  |  |
| 40 | Spectron EF | **CoCrMo** | Reflection XLPE | Lubinus SP II (CrCo) |  |  |  |
| 41 | Spectron EF | **CoCrMo** | Reflection XLPE |  |  |  |  |
| 42 | Spectron EF | **CoCrMo** | Reflection XLPE | Charnley (SS) |  |  |  |
| 43 | Spectron EF | **CoCrMo** | Reflection XLPE |  |  |  |  |
| 44 | Spectron EF | **CoCrMo** | Reflection XLPE |  |  |  |  |
| 45 | Spectron EF | **CoCrMo** | Reflection XLPE | Spectron-EF (CoCrMo) |  |  |  |
| 46 | Spectron EF | **CoCrMo** | Reflection XLPE |  |  |  |  |
| 47 | Spectron EF | **CoCrMo** | Reflection XLPE | Exeter (SS) |  |  |  |
| 48 | Spectron EF | **CoCrMo** | Reflection XLPE | Lubinus SP II (CoCrMo) | NexGen (TiAlV) | NexGen (TiAlV) |  |
| 49 | Spectron EF | **CoCrMo** | Reflection XLPE | MS-30 (SS) |  |  |  |
| 50 | Spectron EF | **Oxinium** | Reflection All-Poly UHMWPE |  |  |  |  |
| 51 | Spectron EF | **Oxinium** | Reflection All-Poly UHMWPE | Spectron-EF (CoCrMo) |  |  |  |
| 52 | Spectron EF | **Oxinium** | Reflection All-Poly UHMWPE | Profile (TiAlV) |  |  |  |
| 53 | Spectron EF | **Oxinium** | Reflection All-Poly UHMWPE | Profile/Tri-lock plus (TiAlV) |  |  |  |
| 54 | Spectron EF | **Oxinium** | Reflection All-Poly UHMWPE | Titan (TiAlV) |  |  |  |
| 55 | Spectron EF | **Oxinium** | Reflection All-Poly UHMWPE | Spectron-EF (CoCrMo) |  |  |  |
| 56 | Spectron EF | **Oxinium** | Reflection All-Poly UHMWPE |  |  |  |  |
| 57 | Spectron EF | **Oxinium** | Reflection All-Poly UHMWPE | Exeter (SS) |  |  | Global Adv. (CoCrMo) |
| 58 | Spectron EF | **Oxinium** | Reflection All-Poly UHMWPE | Charnley (SS) |  |  |  |
| 59 | Spectron EF | **Oxinium** | Reflection All-Poly UHMWPE |  |  |  |  |
| 60 | Spectron EF | **Oxinium** | Reflection All-Poly UHMWPE | Lubinus SP II (CoCrMo) |  |  |  |
| 61 | Spectron EF | **Oxinium** | Reflection All-Poly UHMWPE |  |  |  |  |
| 62 | Spectron EF | **Oxinium** | Reflection All-Poly UHMWPE | Spectron-EF (CoCrMo) |  |  |  |
| 63 | Spectron EF | **Oxinium** | Reflection All-Poly UHMWPE |  |  |  | Delta Xtend (CoCrMo) |
| 64 | Spectron EF | **Oxinium** | Reflection All-Poly UHMWPE | Corail (TiAlV) | Profix (CoCrMo) | NexGen (TiAlV) |  |
| 65 | Spectron EF | **Oxinium** | Reflection All-Poly UHMWPE | Lubinus SP II (CoCrMo) |  |  |  |
| 66 | Spectron EF | **Oxinium** | Reflection All-Poly UHMWPE | Lubinus SP II (CoCrMo) |  |  |  |
| 67 | Spectron EF | **Oxinium** | Reflection XLPE |  |  |  |  |
| 68 | Spectron EF | **Oxinium** | Reflection XLPE | Exeter (SS) | NexGen (TiAlV) |  |  |
| 69 | Spectron EF | **Oxinium** | Reflection XLPE | Spectron-EF (CoCrMo) | LCS (CoCrMo) |  |  |
| 70 | Spectron EF | **Oxinium** | Reflection XLPE |  |  |  |  |
| 71 | Spectron EF | **Oxinium** | Reflection XLPE | Spectron-EF (CoCrMo) |  |  |  |
| 72 | Spectron EF | **Oxinium** | Reflection XLPE | Spectron-EF (CoCrMo) |  |  |  |
| 73 | Spectron EF | **Oxinium** | Reflection XLPE | Charnley (SS) |  |  | Global Adv. (CoCrMo) |
| 74 | Spectron EF | **Oxinium** | Reflection XLPE | Lubinus SP II (CoCrMo) |  |  |  |
| 75 | Spectron EF | **Oxinium** | Reflection XLPE |  | Profix (CoCrMo) |  |  |
| 76 | Spectron EF | **Oxinium** | Reflection XLPE |  |  |  |  |
| 77 | Spectron EF | **Oxinium** | Reflection XLPE | Lubinus SP II (CoCrMo) |  |  |  |
| 78 | Spectron EF | **Oxinium** | Reflection XLPE | Spectron-EF (CoCrMo) |  |  |  |
| 79 | Spectron EF | **Oxinium** | Reflection XLPE |  |  |  |  |
| 80 | Spectron EF | **Oxinium** | Reflection XLPE | Charnley (SS) |  |  |  |
| 81 | Spectron EF | **Oxinium** | Reflection XLPE |  |  |  |  |
