# Supplementary material for: Similarly low blood metal ion levels at 10‐years follow‐up of total hip arthroplasties with Oxinium, CoCrMo, and stainless steel femoral heads. Data from a randomized clinical trial
Source: J Biomed Mater Res B Appl Biomater. 2022 Nov 10;111(4):821–8. doi: 10.1002/jbm.b.35193 (PMC10099800; doi:10.1002/jbm.b.35193)
Supplement: Supplementary file 2 — Appendix S2: Supplement 2. A comparison of the of the blood metal levels (μg/l) in patients with only the study prosthesis (pooled) and those with additional prostheses (pooled). An independent samples Mann–Whitney U test was used to compare ranks. [file JBM-111-821-s002.docx]

**Supplement 2.** A comparison of the of the blood metal levels (μg/l) in patients with only the study prosthesis (pooled) and those with additional prostheses (pooled). An independent samples Mann-Whitney U test was used to compare ranks.

|  | |  |  |  |  |  |
| --- | --- | --- | --- | --- | --- | --- |
| **Metal ion** | **Groups** | | **N** | **Medians**  **(μg/l)** | **Mean ranks** | **p-value** |
| Chromium | Only study prosthesis | | 26 | 0.17 | 40.27 | 0.85 |
|  | Additional prostheses | | 55 | 0.20 | 41.35 |  |
| Cobalt | Only study prosthesis | | 26 | 0.11 | 40.13 | 0.82 |
|  | Additional prostheses | | 55 | 0.14 | 41.41 |  |
| Zirconium | Only study prosthesis | | 26 | 0.07 | 35.58 | 0.15 |
|  | Additional prostheses | | 55 | 0.19 | 43.56 |  |
| Nickel | Only study prosthesis | | 26 | 0.17 | 38.12 | 0.45 |
|  | Additional prostheses | | 55 | 0.20 | 42.36 |  |
